# Parasite infection and the movement of the aquatic snail *Potamopyrgus antipodarum* along a depth cline

**DOI:** 10.1002/ece3.10124

**Published:** 2023-05-29

**Authors:** Frida Feijen, Claudia Buser, Kirsten Klappert, Jukka Jokela

**Affiliations:** ^1^ Eawag, Swiss Federal Institute of Aquatic Science and Technology Dübendorf Switzerland; ^2^ Department of Environmental Systems Sciences, ETH‐Zürich Institute of Integrative Biology Zürich Switzerland

**Keywords:** behavioral manipulation, multiple‐host life cycles, parasitism

## Abstract

1. Parasite species that use two or more host species during their life cycle depend on successful transmission between these species. These successive host species may have different habitat requirements. For example, one host species may be aquatic while the other is terrestrial. To overcome this complicating factor in transmission, a wide diversity of parasite species have adaptations that alter the habitat preference in one host species to facilitate transmission to the next host species.
2. Two common trematode parasites in New Zealand, *Atriophallophorus winterbourni* and *Notocotylus* spp., both have a life cycle with two host species. The aquatic snail *Potamopyrgus antipodarum* is the intermediate host, from which the parasites require transmission to dabbling ducks or other waterfowl. Of these parasites, *A. winterbourni* is most frequently found in snails from the shallow‐water margin. This may indicate parasite‐induced movement of infected snails into the foraging habitat of dabbling ducks.
3. To test whether the parasites manipulate the snails to move into shallow water, we stretched tubular mesh cages across depth‐specific ecological habitat zones in a lake. Both infected and healthy snails were released into the cages. After 11 days, significantly higher infection frequencies of *A. winterbourni* were retrieved from the shallowest end of the cages, while *Notocotylus* spp. frequencies did not vary with depth.
4. The hypothesis that *A. winterbourni* induces its snail host to move into the shallow‐water habitat cannot be rejected based on the experimental results. Although further research is needed to address alternative explanations, the depth preference of infected snails may be due to a parasite adaptation that facilitates trophic transmission of parasites to dabbling ducks.

## Graphical abstract

We provide evidence for behavioral manipulation of the snail *Potamopyrgus antipodarum* by the parasite *Atriophallophorus winterbourni*: infected snails seem to prefer the shallowest habitat. We were interested in movement across littoral habitat zones, and in order to test this, we designed cages that span a depth gradient in the lake. This allows for the investigation of movement patterns of snails in as natural a setting as possible.

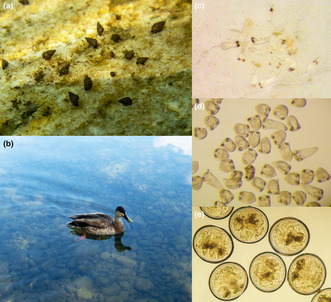

## 1 INTRODUCTION

Understanding the transmission of parasites between hosts is central to studies on epidemiology, host–parasite co‐evolution, and the evolution of complex life cycles in parasites. Parasites with complex life cycles require transmission between two or more host species to complete a full life cycle (Galaktionov & Dobrovolskij, 2003). Parasitic adaptations to facilitate transmission between different host species have gained interest since the work by Holmes and Bethel (1972) on behavioral manipulation. Adaptations may include behavioral manipulation or modifications to the appearance of a host to make it more conspicuous (Holmes & Bethel, 1972; Poulin, 1995; Thomas et al., 2005). When successive hosts have different habitat requirements, parasites may have evolved the ability to induce migration of their host to an area where transmission to the next host species is more likely. Such adaptations have been demonstrated for a wide diversity of parasites, including acanthocephalans, platyhelminths, and nematomorphs (Bakker et al., 2017; Curtis, 1987; Helluy, 1984; Holmes & Bethel, 1972; Thomas et al., 2002, 2005).

After an initial increase in interest in behavioral manipulation by parasites, a debate began on whether changes in host behavior are true parasite adaptations (Poulin, 1995, 2000; Poulin & Maure, 2015; Thomas et al., 2005). When infected hosts display behavior that deviates from that of uninfected hosts, this can be due to a variety of reasons. A behavioral modification may be beneficial to the host, rather than to the parasite. It may also be a side effect of infection that has no advantage for either the host or the parasite. The different behavior may have been present prior to infection and may have predisposed the host to exposure. To determine whether behavioral changes are parasite adaptations, it is necessary to explore such alternative hypotheses, separate cause and effect and it must be shown that the trait increases parasite fitness (Poulin & Maure, 2015).

**FIGURE 1:**
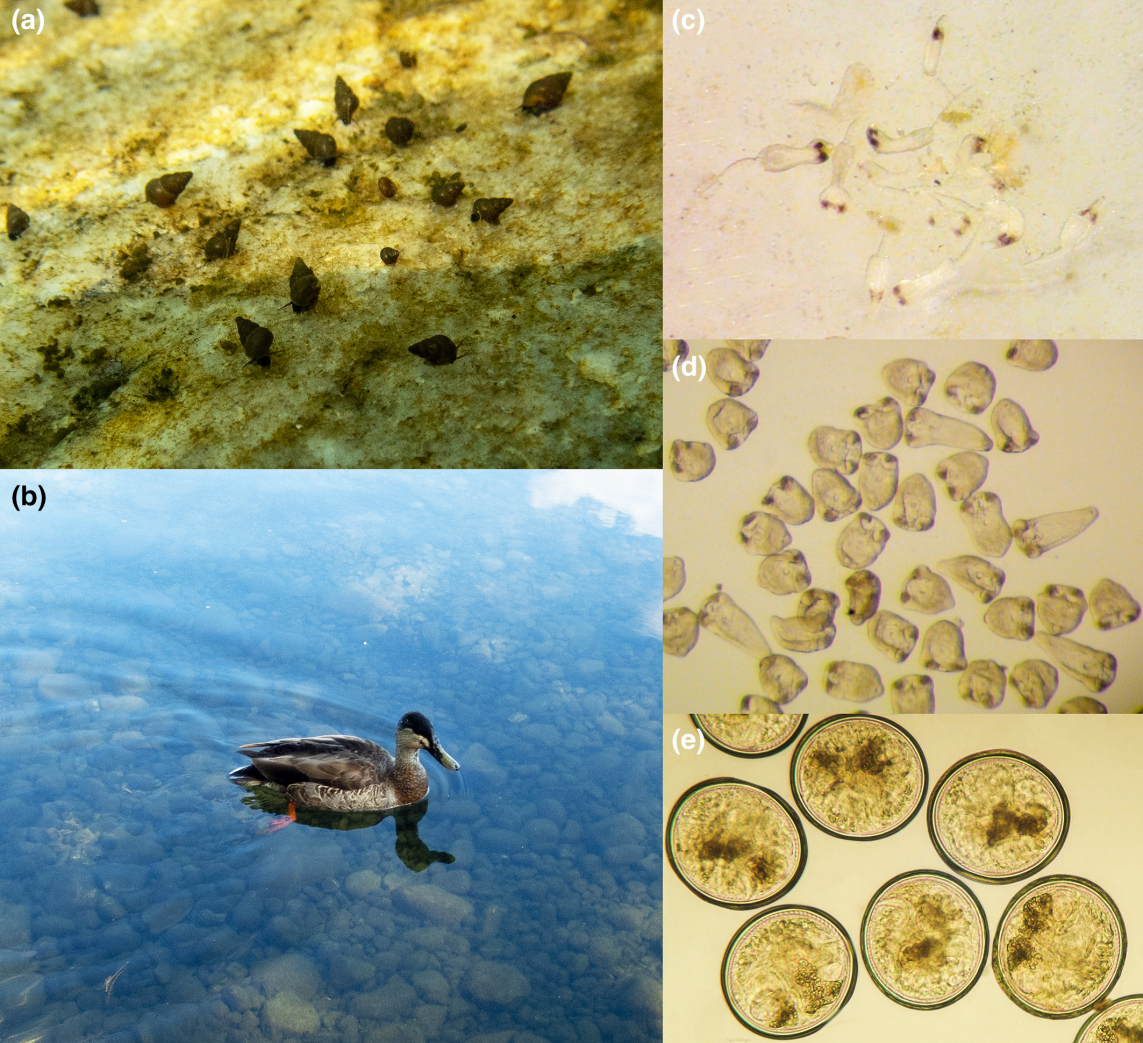
An overview of the study organisms. (a) The snail *Potamopyrgus antipodarum* is the intermediate host for the trematode parasites. (b) A dabbling duck at Lake Alexandrina, New Zealand, an important definitive host species. (c) Free‐swimming cercariae of *Notocotylus* spp., as extracted from a snail. (d) Adult worms of *Atriophallophorus winterbourni* as they would be found in the gut of waterfowl, here in a laboratory culture following the protocols described in Blasco‐Costa et al. (2019). (e) Metacercariae of *A. winterbourni*, as extracted from a snail.

The New Zealand trematodes *Atriophallophorus winterbourni* (formerly referred to as *Microphallus* sp.) and *Notocotylus* spp. (Figure 1) both use the freshwater snail *Potamopyrgus antipodarum* as an intermediate host and waterfowl such as dabbling ducks as a definitive host (Blasco‐Costa et al., 2019; Hechinger, 2012; Lively & McKenzie, 1991; Osnas & Lively, 2011; Winterbourn, 1974). Since both *Atriophallophorus* and *Notocotylus* belong to the order Plagiorchiida, the parasite eggs most likely require passive ingestion by the snail host (Galaktionov & Dobrovolskij, 2003). Maturation of the parasitic infection in the snails takes more than 3 months for *A. winterbourni* (Lively & McKenzie, 1991). The cercariae encyst within the gonad of the snail and the life cycle can only continue after the snail has been ingested by the definitive host. In contrast, *Notocotylus* spp. infections have swimming cercariae that leave the snail host and encyst on vegetation and often on the outer shell of the host. Both the ingestion of plants and infected snails may lead to the successful transmission of these parasites to waterfowl. Cercarial shedding of *Notocotylus* spp. was found to begin in October for snails that were exposed in May (Bisset, 1977). Both parasite species reproduce sexually in the gut of the bird and the parasite eggs are shed with the feces. Osnas and Lively (2011) found that *Notocotylus* spp. were associated with diving ducks (New Zealand Scaup) while *A. winterbourni* was associated with either dabbling ducks or diving ducks. However, the sample sizes in this study were relatively small (*N* = 4 and 13 for dabbling and diving ducks, respectively) to draw conclusions about associations between definitive hosts and parasites.

**FIGURE 2:**
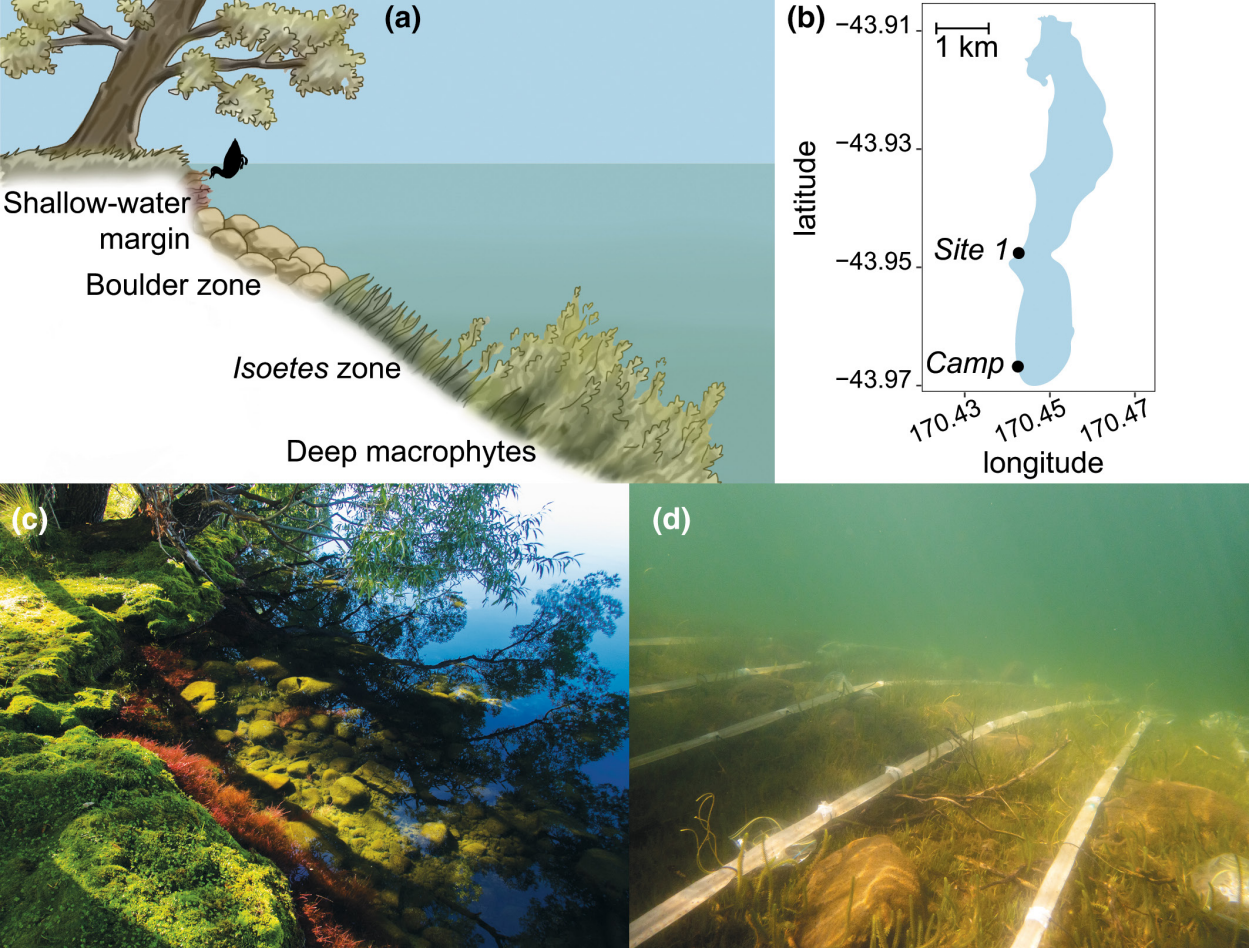
Habitat zonation, experimental cages, and sites at Lake Alexandrina, New Zealand. (a) Habitat zonation with depth. The shallow‐water margin consists of a small vertical drop and is characterized by willow roots and moss (ca. 0.0‐ to 0.5‐m depth). The adjacent boulder zone lacks dense vegetation due to wave action (ca. 0.5‐ to 1.5‐m depth). Dense mats of *Isoetes alpinus* are found between ca. 1.5‐ and 3.0‐m depth. Tall macrophytes are found below ca. 3.0‐m depth. (b) The sites at Lake Alexandrina, as specified in the text (*Camp* and *Site 1*). (c) The shallow‐water margin and the adjacent boulder zone, as seen from the shore. (d) To test whether the parasites *Atriophallophorus winterbourni* and *Notocotylus* spp. induce the movement of the snail host *Potamopyrgus antipodarum* towards the shallow‐water margin, snails were released in cages that were stretched across a depth cline from the base of the willow roots up to the beginning of the macrophyte zone (0.8–2.8 m).

In the shallow‐water margin (<50 cm depth; see Figure 2a) of Lake Alexandrina, New Zealand, a large proportion of snails are infected by *A. winterbourni* (Vergara et al., 2013). Although the prevalence of infection in the shallow‐water margin varies over time and by location, it can occasionally exceed 50%. In deeper habitat zones, the prevalence is typically well below 10% (Jokela & Lively, 1995; Vergara et al., 2013). This could be the result of a higher risk of exposure in shallow habitat, but an alternative explanation for this pattern could be that the parasite induces movement across a depth cline. Such a behavioral modification may facilitate the transmission of the parasite to dabbling ducks. The foraging probability by dabbling ducks decreases sharply with depth and the foraging should not normally exceed depths of 50 cm, since these birds feed from the surface (Arzel & Elmberg, 2004; Behney, 2014; Guillemain et al., 2000). Earlier studies have shown that infected snails from natural populations spend more time foraging on the exposed upper side of rocks, during the time of day when waterfowl are most actively foraging (Levri, 1998a, 1998b; Levri & Lively, 1996). This behavioral modification is maintained even when the food source is removed from the rocks, suggesting that it is not due to an increased energetic demand by the parasite (Levri, 1999). Both phototaxis and geotaxis were tested as possible mechanisms behind this observation and the latter was found to be influenced by infection status (Levri et al., 2007; Levri & Fisher, 2000).

For *Notocotylus* spp. there is currently no evidence that it might manipulate the behavior of its snail host. It could be hypothesized that host manipulation is not needed because the free‐swimming cercariae may already shorten the distance to the definitive host. However, this generalization has been shown not to hold in the past, since parasites with free‐swimming cercariae may still modify host behavior (Curtis, 1987). Alternatively, it could be hypothesized that trophic transmission is disadvantageous for *Notocotylus* spp., since these infections cation of the snail bn shed cercariae for up to 8 months (Bisset, 1977). Ingestion of the snail by a definitive host may reduce its fitness by decreasing the lifetime cercarial output, even when many cercariae encyst on the shell of the snail.

To test the hypothesis that *A. winterbourni* induces snail movement across a depth cline, we designed an in‐situ experiment in which both infected and uninfected snails were released at the deep ends of 5 m long, tubular mesh cages that were stretched across a depth cline (0.8–2.8 m). After sufficient time passed for the snails to disperse within the cages, we assessed how infection frequencies with *A. winterbourni* and *Notocotylus* spp. varied with depth. While the frequency of infections by *Notocotylus* spp. was the same for all cage sections, we found that snails infected by *A. winterbourni* were most common in the shallowest part of each cage. This finding suggests that the snails' habitat choice along a depth cline is affected by *A. winterbourni* and that infected snails move across littoral habitat zones toward the shallow‐water margin.

## 2 MATERIALS AND METHODS

### 2.1 Study site

Lake Alexandrina is a sub‐alpine lake that lies east of the Southern Alps in New Zealand. The habitat zonation in the lake is dependent on wave action and depth (light penetration), while the local width of each habitat zone depends on the steepness of the lake bed (Jokela & Lively, 1995; Ward & Talbot, 1984). Figure 2a shows a detailed outline of the habitat zonation.

### 2.2 Cage experiment

To test for behavioral manipulation of snails by trematodes across an environmental gradient in Lake Alexandrina, 10 tubular mesh cages, each 5 m in length, were constructed and placed side by side in the lake (Figure 2d). The experiment was conducted in the summer of 2018. After placing the shallow end at the bottom of the vertical shore bank, at a depth of 0.8 m, the deep end of the cage reached 2.8‐m depth. The 10 cages were secured with sandbags at each end and one in the middle. Cages were made from 500‐micron mesh tubing with a 3.5 cm diameter (Sefar AG) and prepared to be sealed at five 1‐m intervals. The cages were strengthened with a structural backbone on the sediment side, while tension from untightened cable tie loops maintained the tubular structure at 33 cm intervals. The empty cages were installed in the water at *site 1* (Figure 2b) on the 10th of January, with both ends closed. The cages were left for 10 days to develop a biofilm for snails to forage on, before the experiment started.

In order to obtain a sample of snails with a high prevalence of infection, snails were collected on the 19th of January from the shallow‐water margins at two sites (*Camp* & *Site 1*, Figure 2b), where high infection frequencies have been consistently reported (Vergara et al., 2013). Snails from both sites were distributed across 11 sets of equal volumes. Each set contained approximately 800 snails of which 39% originated from *Camp* and 61% originated from *Site 1*. For the eleventh set, snails were kept separate by the site of origin and functioned as a control to determine the background infection prevalence and the relative contribution of each source site to the infections in each of the experimental cages. During the experimental period, these controls were kept in a 1.5‐L mesh (250 μm) cage in the water at the experimental site. After the experiment was terminated, 100 randomly selected snails were dissected from each of these two controls.

On the 20th of January, one volume of snails from each site was released into the deep end of each of the 10 cages. As infected snails are known to move more slowly than uninfected snails (Levri & Fisher, 2000), a release in the deep end is preferred to a release in the shallow end because the latter method may artificially lead to aggregations of slow ‐moving snails in the shallow end. Before releasing the snails, five randomly selected cages were turned 180° to reverse the depth gradient in the biofilm (food resource). After 1 day, snails could already be seen in the upper sections of the cages, indicating that snails would have ample time to disperse throughout the cages during the 11‐day experimental period.

On the 31st of January, in anticipation of a storm, we effectively ended the experiment by sealing the cages at 1‐m intervals to preserve the distribution of snails across the five sections. The sealing of the 1‐m sections was done by free divers to ensure that the cages were not disturbed and the snails were trapped in position. The cages were then moved to deeper water to protect them from the wave action during the storm. Both the experimental and control cages were retrieved from the deeper water on the 4th of February. A construction flaw was discovered in one of the turned cages, so this replicate was dismissed. Snails were transported to the laboratory in Switzerland (Eawag, Dübendorf), where randomly selected snails were dissected from the shallow, middle, and deep sections of each of the nine remaining cages between the 12th and 17th of February (*N* = 50 for all sections, with the exception of *N* = 70 and 91 for two of the deep sections). Infection status by both *A. winterbourni* and *Notocotylus* spp. was recorded to estimate parasite prevalence in each sample. Juvenile snails smaller than 2.5 mm were not included. The period from the beginning of the experiment (January 20–February 17) until the end of the experiment was short enough to exclude exposure during the experiment as a significant factor, since infections take several months to develop (Bisset, 1977; Krist et al., 2000; Lively & McKenzie, 1991).

### 2.3 Statistical analysis

To test whether the prevalence of infection by either *A. winterbourni* or *Notocotylus* spp. differed between sections of the cages, separate mixed‐effects logistic regression models with logit link function were fitted for each infection type using R version 4.0.5 (R Core Team, 2021). In these models, the infection status of individual snails was treated as binary response variable. We used section depth, treatment (turned or not turned), and the interaction between depth and treatment as explanatory fixed factors, while cage identity was treated as a random effect. As snails were only dissected from the shallowest, middle, and deepest of the five sections of each cage, we treated depth as a categorical fixed factor. Three snails were co‐infected by both *Notocotylus* spp. and *A. winterbourni*. These co‐infected snails were included in both models as infected individuals, since their frequency was too low to confound the analyses. An initial model fit with the R package lme4 1.1‐27.1 (Bates et al., 2015) led to singularity. We therefore fitted the model with the R package blme 1.0‐5 (Chung et al., 2013), which implements a maximum penalized likelihood approach to handle singularity. Analysis of deviance tables (Type III Wald *χ* ^2^ test) was calculated using the R package car 3.0‐11 (Fox & Weisberg, 2019).

### 2.4 Distribution of *A. winterbourni* in cages

Since only a random subset of snails was dissected from the deepest, middle, and shallowest sections of the five sections of each cage, we were interested in projecting what fraction of the snails infected by *A. winterbourni* traveled to the shallowest section in each cage. First, the number of infected snails in each shallow section was calculated by multiplying the number of snails in each of the nine shallow sections with the corresponding prevalence of infection. Then, in order to estimate the total number of *A. winterbourni* infections in each cage, we multiplied the background infection prevalence of the experimental snails with the total number of snails in each cage. The background infection prevalence was determined by multiplying the prevalence from each of the two sites by their contribution to the total number of snails in each cage. Experimental snails that were not dissected were counted by analyzing photos of the snails in a white tray in ImageJ 1.52a (Schneider et al., 2012). The watershed function was used to separate clusters of snails before an automated final count. The same minimum size limit of 2.5 mm that was used during the dissections, was also used for the snail counts from the photographs.

## 3 RESULTS

**FIGURE 3:**
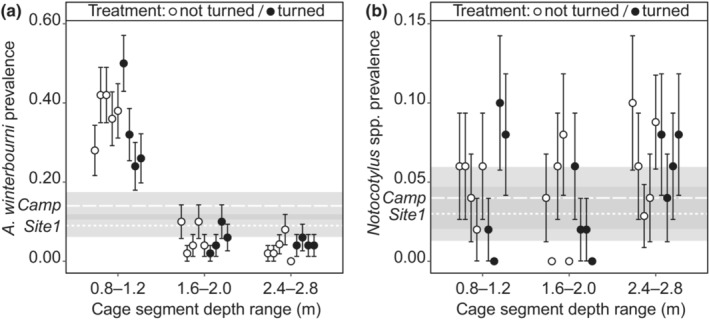
The high prevalence of *Atriophallophorus winterbourni* infections in the shallowest section of nine experimental cages indicates behavioral manipulation (a), while the prevalence of *Notocotylus* spp. infection is equal across the cages (b). Four cages were turned to test for an effect of depth‐specific biofilm development. Dashed horizontal lines show the background infection prevalence of the two source sites for snails, error bars and gray shading show the binomial standard error of prevalence (a, b).

We found the highest prevalence of infection with *A. winterbourni* in the shallowest (0.8‐ to 1.2‐m depth) 1‐m section of the cages (*χ* ^2^ = 107.49, df = 2, *p* < 2 × 10^−16^; Table 1 and Figure 3a). On average, the prevalence of infection in this section was 33% in the turned cages and 37% in the nonturned cages (Figure 3a). By contrast, the prevalence of *A. winterbourni* was much lower in the middle 1‐m section (1.6‐ to 2.0‐m depth, both cage treatments: 6%) and in the deepest 1‐m section (2.4‐ to 2.8‐m depth; turned: 5%; not turned: 3%). The prevalence of *A. winterbourni* infections was not affected by either the turning treatment or the interaction term between the turning treatment and depth (Table 1). *A. winterbourni* infections that were not at transmission stage (*N* = 4), were only found in the deepest cage section. There was no significant difference in the prevalence of snails infected by *Notocotylus* spp. between any of the depth‐specific cage sections (*χ* ^2^ = 2.32, df = 2, *p* = .31; Table 1 and Figure 3b).

**TABLE 1:** Analysis of deviance table (Type III Wald *χ* ^2^ test; mixed‐effects logistic regression models with logit link function) for the cage experiment analysis.

Binomial response variable	Intercept/explanatory variables	*χ* ^2^	df	*p*
*Atriophallophorus winterbourni* infection status	Intercept	102.756	1	<2 × 10^−16^
	Depth (factor)	107.490	2	<2 × 10^−16^
	Turning treatment	0.836	1	.361
	Interaction term	1.502	2	.472
*Notocotylus* spp. infection status	Intercept	111.647	1	<2 × 10^−16^
	Depth (factor)	2.318	2	.314
	Turning treatment	0.001	1	.983
	Interaction term	0.406	2	.816

*Note*: The cage experiment indicates parasite‐induced host migration toward the shallow‑water margin for the trematode *Atriophallophorus winterbourni* but not for *Notocotylus* spp., since only the prevalence of *A. winterbourni* infections changes significantly with the depth of the cage section. The analysis includes the infection status of 1411 snails as binomial response variable. The three depth‐specific sections of each of the nine cages and the turning treatment (four turned, five not turned) were treated as categorical explanatory variables. In addition, the interaction term between section depth and turning treatment was included. The variance explained by cage (random effect) was 0.03 ± 0.17 (standard deviation) for the *A. winterbourni* model and 0.05 ± 0.23 for the *Notocotylus* spp. model.

The background infection prevalence of *A. winterbourni* among experimental snails was 11%. More precisely, the prevalence was 14% for *Camp* and 9% for *Site 1*. These sites accounted for 39% and 61% of the experimental snails, respectively. The prevalence of *A. winterbourni* infections in the shallowest cage sections was 35% on average, which was more than three times higher than the background prevalence of 11% and exceeded the background prevalence of both source sites (Figure 3a). The prevalence in the shallow sections was projected to correspond to 89% of the infections that entered the experiment. By contrast, only 27% of the total number of snails was retrieved from the shallowest sections.

## 4 DISCUSSION

Parasite traits that facilitate transmission should be favored by natural selection (Holmes & Bethel, 1972; Thomas et al., 2005). For example, when successive host species have different habitat requirements, a parasite may benefit from the ability to induce the movement of its current host towards the habitat of the next host (Holmes & Bethel, 1972; Thomas et al., 2005). In Lake Alexandrina (New Zealand), infections by *A. winterbourni* are most common in the shallow‐water margin (Vergara et al., 2013). Two possible reasons for this observation are (I) a higher risk of exposure in the shallow‐water margin or (II) a parasite‐induced preference for the shallow‐water margin. When we placed both infected and uninfected snails in tubular cages that were stretched across a littoral depth gradient in the lake, we found that snails infected with *A. winterbourni* were mostly retrieved from the shallowest section of cages. Based on this result, the hypothesis that infected snails are induced to move toward the shallow‐water margin by the parasite cannot be rejected. The high prevalence of *A. winterbourni* in the shallow‐water margin may thus be the result of a parasite adaptation to facilitate transmission, similar to that found in several other host–parasite systems (Curtis, 1987; Helluy, 1984; Holmes & Bethel, 1972; Thomas et al., 2002). In future research, it will be important to estimate the risk of exposure between habitats and to investigate how infection affects the behaviour of snails from deeper habitat zones.

Adaptive behavioral manipulation by parasites—which entails a higher fitness for manipulating parasites relative to conspecifics that do not exhibit the trait—needs to be verified by examining any alternative explanations (Poulin, 1995; Poulin & Maure, 2015; Thomas et al., 2005). For example, behavioral changes in infected hosts could be due to host adaptations that mitigate parasite virulence, they could be behavioral traits that predisposed the host to contracting an infection, or they could be side‐effects of infection that provide no benefit to either the host or the parasite (Poulin, 1995; Poulin & Maure, 2015; Thomas et al., 2005).

Firstly, the movement of infected *P. antipodarum* snails to shallow habitat is very unlikely to be a case of host adaptation to parasitism, since *A. winterbourni* sterilizes the snail and selection cannot act on host traits post‐infection. Secondly, snails with a preference for the shallowest habitat may be more prone to infection if the risk of infection is highest in the shallow‐water margin. Although the variation in exposure risk with depth still needs to be assessed, one counter‐argument for this explanation is that all the snails with early‐stage infections were found only in the deepest cage sections (*N* = 4). Further experiments could be performed with experimentally exposed snails from different habitats to clearly distinguish cause and effect, or by caging snails at various depths to test for habitat‐specific risk of infection. Thirdly, infected snails may have an increased foraging need due to energetic demands of the parasite. This could lead them to prefer biofilms in the shallow habitat, but we found no effect on cage turning. However, it should be noted that the algal growth during the experimental phase may have reduced the effect of the initial incubation phase of the cages. Furthermore, if an increased need for foraging was driving the aggregation in the shallow, the same would be expected for snails that are infected by *Notocotylus* spp., since these snails are more susceptible to starvation than those infected by *A. winterbourni* (Jokela et al., 1999). In addition to food availability, other environmental factors to consider include the dissolved oxygen concentration and water temperature. Both of these may vary slightly with depth, even though Lake Alexandrina is not stratified (Ward & Talbot, 1984). Finally, snails infected by *A. winterbourni* are not likely to have aggregated in the shallow end of the cages ahead of the healthy snails because they move faster than healthy snails, since infection reduces snail speed (Levri & Fisher, 2000).

For closely related parasites, behavioral manipulation of hosts may be an ancestral trait (Hansen & Poulin, 2005; Moore, 1984). Apart from *A. winterbourni*, the vertical distribution of hosts in the aquatic environment is affected by at least two other trematodes in the Microphallidae family: *Gynaecotyla adunca* and *Microphallus papillorobustus*, but it is unlikely to be ubiquitous for the family (Curtis, 1987; Hansen & Poulin, 2005; Helluy, 1984). The three species that induce vertical migration differ considerably in their mode of transmission, their life cycle, and in the identity of the affected host. The crustacean *Gammarus insensibilis* is manipulated by *M. papillorobustus* to increase trophic transmission to birds (Helluy, 1984), while the estuarine snail *Ilyanassa obsoleta* is manipulated by *G. adunca* to enhance transmission of free‐moving cercarial larvae to semi‐terrestrial crustaceans (Curtis, 1987). Interestingly, one of the most highly duplicated gene families in *A. winterbourni* involves the glutamine synthase pathway, which has been implicated in behavioral modification by *M. papillorobustus* (Helluy & Thomas, 2010; Zajac et al., 2021). Wider taxon sampling and phylogenetic trait mapping will be required to understand the evolution of adaptive host manipulation in Microphallids.

Two very closely related species of *Atriophallophorus* co‐exist in the same lakes in New Zealand and both use *P. antipodarum* as intermediate host, but the relative frequency of these species changes with depth. For example, 95% of the infections in the shallow‐water margin of Lake Alexandrina were found to be *A. winterbourni* (where we collected the snails for the current study) while the—so far undescribed—*Atriophallophorus* sp. was more frequent than *A. winterbourni* in deeper habitat zones (Feijen et al., 2022). This could be explained if *A. winterbourni* manipulates the host to travel to shallow water while *Atriophallophorus* sp. lacks the trait and may rely on diving ducks as a definitive host, rather than dabbling ducks. Selective pressures from alternative definitive host species with different feeding habits may be considered as a driver of parasite speciation. The New Zealand *Atriophallophorus* species would thus be of interest for further research on behavioral manipulation by parasites.

The use of tubular mesh cages across the environmental cline was found useful to test our hypothesis on behavioral manipulation in a natural setting. This provided new evidence for behavioral differences between healthy *P. antipodarum* snails and those infected by the parasite *A. winterbourni*. Further research is needed, especially as the current study focuses only on naturally infected snails from the shallow‐water margin and it does not address variation in exposure risk between habitats. However, our finding complements the earlier work by Levri et al. on host behavioral manipulation by *A. winterbourni*. It is possible that the parasite *A. winterbourni* induces host movement towards the shallow‐water margin to facilitate trophic transmission to dabbling ducks.

## AUTHOR CONTRIBUTIONS

**Frida Feijen:** Conceptualization (lead); data curation (lead); formal analysis (lead); investigation (lead); methodology (lead); visualization (lead); writing – original draft (lead); writing – review and editing (lead). **Claudia Buser:** Methodology (supporting); writing – review and editing (supporting). **Kirsten Klappert:** Methodology (supporting); writing – review and editing (supporting). **Jukka Jokela:** Formal analysis (supporting); funding acquisition (lead); methodology (equal); supervision (supporting); writing – review and editing (supporting).

## FUNDING INFORMATION

This project was funded by the Swiss National Science Foundation grant 31003A_166667/1 and ETH Grant ETH‐36 15‐2, both awarded to JJ.

## CONFLICT OF INTEREST STATEMENT

The authors declare no competing interests.

### OPEN RESEARCH BADGES

This article has earned an Open Data badge for making publicly available the digitally‐shareable data necessary to reproduce the reported results. The data is available at [https://doi.org/10.5061/dryad.bzkh189f6].

## Data Availability

The data used for this study have been deposited on dryad: https://doi.org/10.5061/dryad.bzkh189f6.
